# Identification and characterization of a novel aminoglycoside *O*-nucleotidyltransferase ANT(6)-If from *Paenibacillus thiaminolyticus* PATH554

**DOI:** 10.3389/fmicb.2023.1184349

**Published:** 2023-06-29

**Authors:** Junwan Lu, Yuning Sha, Mengdi Gao, Weina Shi, Xi Lin, Kewei Li, Qiyu Bao, Chunlin Feng

**Affiliations:** ^1^Medical Molecular Biology Laboratory, School of Medicine, Jinhua Polytechnic, Jinhua, China; ^2^Key Laboratory of Medical Genetics of Zhejiang Province, Key Laboratory of Laboratory Medicine, School of Laboratory Medicine and Life Sciences, Institute of Biomedical Informatics, Ministry of Education, Wenzhou Medical University, Wenzhou, China

**Keywords:** *Paenibacillus thiaminolyticus*, aminoglycoside-modifying enzyme, comparative genomics, enzyme kinetic analysis, *ant(6)-If*

## Abstract

**Background:**

*Paenibacillus thiaminolyticus*, a species of genus *Paenibacillus* of the family *Paenibacillaceae*, exists widely in environments and habitats in various plants and worms, and occasionally causes human infections. This work aimed to characterize the function of a novel aminoglycoside *O*-nucleotidyltransferase resistance gene, designated *ant(6)-If*, from a *P. thiaminolyticus* strain PATH554.

**Methods:**

Molecular cloning, antimicrobial susceptibility testing, enzyme expression and purification, and kinetic analysis were used to validate the function of the novel gene. Whole-genome sequencing and comparative genomic analysis were performed to investigate the phylogenetic relationship of ANT(6)-If and other aminoglycoside *O*-nucleotidyltransferases, and the synteny of *ant(6)-If* related sequences.

**Results:**

The recombinant with the cloned *ant(6)-If* gene (pMD19-*ant(6)-If*/DH5α) demonstrated a 128-fold increase of minimum inhibitory concentration level against streptomycin, compared with the control strains (DH5α and pMD19/DH5α). The kinetic parameter *k*_cat_*/K*_m_ of ANT(6)-If for streptomycin was 9.01 × 10^3^ M^−1^·s^−1^. Among the function-characterized resistance genes, ANT(6)-If shared the highest amino acid sequence identity of 75.35% with AadK. The *ant(6)-If* gene was located within a relatively conserved genomic region in the chromosome.

**Conclusion:**

*ant(6)-If* conferred resistance to streptomycin. The study of a novel resistance gene in an unusual environmental bacterium in this work contributed to elucidating the resistance mechanisms in the microorganisms.

## Introduction

*Paenibacillus* is a genus of firmicute in the family *Paenibacillaceae*. The genus is characterized by aerobic or facultatively anaerobic, oval-shaped, endospore-forming, Gram-positive, -negative or-variable *bacillus* (Sáez-Nieto et al., 2017), and it contains more than 200 recognized species.^1^ *Paenibacillus* has been isolated from a wide range of sources and produces a variety of antibiotics, enzymes, and exopolysaccharides that are beneficial to medicine, the manufacturing industry, and bioremediation. On the negative aspect, some *Paenibacillus* species can infect honeybees and cause dairy spoilage, and occasionally act as an opportunistic pathogen in human beings (Grady et al., 2016). *Paenibacillus thiaminolyticus* was initially included in the genus *bacillus* and was reclassified into the genus *Paenibacillus* based on the results of 16S rRNA gene and cellular fatty acid composition analyses (Shida et al., 1997). *Paenibacillus thiaminolyticus* can produce thiaminase I enzyme (Richter et al., 2009), antimicrobial peptide polymyxin A1 and paenibacterin that are active against Gram-negative and Gram-positive pathogens (Huang and Yousef, 2014; Wu et al., 2022). Infections caused by clinical *P. thiaminolyticus* isolates have also been reported (Ouyang et al., 2008; Di Micco et al., 2021; Morton et al., 2022).

Aminoglycoside antibiotics were first isolated from bacteria *Streptomyces* and *Micromonospora* and showed effective treatment of infections caused by Gram-negative and some Gram-positive bacteria. But bacterial resistance was observed more frequently as the widespread use of these antibiotics in clinical practice and the remarkable ability of bacteria to develop resistance to antibiotics (Becker and Cooper, 2013). Resistance mechanism of aminoglycoside antibiotics includes modification of aminoglycoside antibiotics by aminoglycoside-modifying enzymes (AMEs), increased efflux pumps or decreased permeability of the bacterial outer membrane, biofilm formation, methylation of 16S rRNA ribosomal subunit etc. (Wang et al., 2022). Among the various mechanisms, the most common resistance mechanism is either intrinsic or acquired ability to produce AMEs (Wachino et al., 2020). Enzymatic modification at −OH or − NH_2_ groups of the 2-deoxystreptamine nucleus or the sugar moieties in aminoglycoside drugs can be accomplished by *N*-acetyltransferases (AACs), *O*-adenyltransferases (ANTs) and *O*-phosphotransferases (APHs; Ramirez and Tolmasky, 2010). AMEs are classified by different sites of modification. For instance, ANT(6) catalyzes nucleotidylylation of streptomycin at the hydroxyl group at position 6, and ANT(9) catalyzes nucleotidylylation of spectinomycin at the hydroxyl group at position 9. Genes coding for ANT(6) have been named *ant(6)-Ia*, *ant(6)-Ib*, and *aadK* etc. (Ramirez and Tolmasky, 2010; Hormeño et al., 2018).

In this work, with molecular cloning, whole genome sequencing and enzyme kinetic analyses, we described a novel aminoglycoside resistance gene *ant(6)-If* encoded on the chromosome of *P. thiaminolyticus* isolated from an animal fecal swab sample.

## Materials and methods

### Bacterial strains and plasmids

The bacteria and plasmids used in this study were listed in Table 1. *P. thiaminolyticus* PATH554 was isolated from the fecal swab sample of a rabbit during a survey on the antimicrobial resistance of bacteria from livestock in a farm in Wenzhou, China. Taxonomic classification of PATH554 included 16S rRNA gene homology, digital DNA–DNA hybridization (dDDH; Meier-Kolthoff et al., 2013), genome distance estimation (Ondov et al., 2016), and whole-genome average nucleotide identity (ANI) analyses (Richter and Rosselló-Móra, 2009).

**Table 1:** Bacteria and plasmids used in this study.

Strain and plasmid	Description	Reference
PATH554	The wild-type strain of *P. thiaminolyticus* PATH554	This study
DH5α	*E. coli* DH5α as a host for cloning of the *ant(6)-If* gene	Our laboratory collection
BL21	*E. coli* BL21 as a host for expression of the *ant(6)-If* gene	Our laboratory collection
ATCC 29213	*S. aureus* ATCC 29213 as the quality control for antimicrobial susceptibility testing	Our laboratory collection
pMD19-*ant(6)-If*/DH5α	DH5α carrying the recombinant plasmid pMD19-*ant(6)-If*	This study
pCold I-*ant(6)-If*/BL21	BL21 carrying the recombinant plasmid pCold I-*ant(6)-If*	This study
pMD19	Cloning vector for the *ant(6)-If* gene with its upstream promoter region, AMP^r^	Our laboratory collection
pCold I	Expression vector for the ORF of the *ant(6)-If* gene, AMP^r^	Our laboratory collection

AMP^r^: ampicillin resistance.

### Antibiotic susceptibility testing

All the antimicrobials were bought from a pharmacy or a hospital. The antimicrobial agents tested in this work were listed in Table 2, including gentamicin, tobramycin, neomycin, streptomycin, sisomicin, ribostamycin, amikacin, spectinomycin, kanamycin, and paromomycin. The minimum inhibitory concentrations (MICs) were determined with the agar dilution method following the Clinical and Laboratory Standards Institute antimicrobial susceptibility testing standard M100 (31st Edition, 2021).

**Table 2:** MICs (μg/mL) of *Paenibacillus thiaminolyticus* PATH554 and the cloned *ant(6)-If* gene.

	ATCC 29213	PATH554	DH5α	pMD19/DH5α	pMD19-*ant(6)-If*/DH5α
Gentamicin	0.125	1	1	1	<0.25
Tobramycin	0.125	16	0.5	0.5	0.25
Neomycin	1	<1	2	2	<1
Streptomycin	4	32	4	4	512
Sisomicin	2	1	<0.5	<0.5	<0.5
Ribostamycin	<2	64	4	2	2
Amikacin	<2	8	2	2	1
Spectinomycin	64	16	8	8	8
Kanamycin	<2	64	2	2	1
Paromomycin	1	4	4	4	2

### Cloning of the *ant(6)-If* gene

Primers were designed using SnapGene.^2^ The open reading frame (ORF) of the *ant(6)-If* gene and its promoter region were amplified by PCR with the primers listed in Table 3 and ligated into the T-Vector pMD19 with the T4 DNA ligase (Takara Bio, Inc., Dalian, China). Then the recombinant plasmid was transformed into *Escherichia coli* DH5α using the calcium chloride method and cultured on Luria-Bertani agar plates supplemented with 100 μg/mL ampicillin. The sequence of the cloned insert was verified by PCR and sequencing (Shanghai Sunny Biotechnology Co., Ltd., Shanghai, China).

**Table 3:** Primers used to clone the *ant(6)-If* gene.

Primer^a^	Sequence (5′ → 3′)^b^	Restriction endonuclease	Vector	Annealing temperature (°C)	Amplicon size (bp)
pro-*ant(6)-If*-F	GGGCAATGGTTTCGTTACTGAGAAG		pMD19	60	1,096
pro*-ant(6)-If*-R	GCGCAAAAATATAACCGCCCACTAA		pMD19		1,096
orf-*ant(6)-If*-F	CGGGATCCCTGGTGCCGCGCGGCAGCTTGAGAAATGAACAAGAAATGATGAAC	*Bam*HI *+* Thrombin	pCold I	60	889
orf-*ant(6)-If*-R	CCAAGCTTTCACTTCCATGAATTTCGAATATG	*Hind*III	pCold I		889

a Primers started with “pro” were used to clone the *ant(6)-If* gene with its promoter region; primers started with “orf” were used to clone the ORF of the *ant(6)-If* gene.

b The underlined sequences indicated the restriction endonuclease sites.

### Expression and purification of the ANT(6)-If enzyme

The ORF of the *ant(6)-If* gene was amplified by PCR with the primers listed in Table 3 and ligated into the pCold I vector with the cleavage sites of thrombin, restriction endonuclease *Bam*HI and *Hind*III. The recombinant plasmid was transformed into *E. coli* BL21. Then ANT(6)-If was overexpressed in *E. coli* BL21/pCold I-*ant(6)-If* and purified as described previously (Qing et al., 2004). When the OD_600_ of the culture reached 0.6–0.8 at 37°C, the expression of ANT(6)-If protein was inducted by adding 1 M isopropyl-β-d-thiogalactoside, and additional cultivation for 20 h at 16°C. Bacteria were collected by centrifugation (8,000 × g, 10 min) at 4°C and resuspended in lysis buffer (20 mM Tris–HCl, 150 mM NaCl, 3 mM β-mercaptoethanol, 0.5% Nonidet-P-40, pH 8.0). Then bacteria were disintegrated by sonication, and the debris was removed by centrifugation (12,000 × g, 30 min) at 4°C. The lysates were incubated with pre-equilibrated nickel-nitrilotriacetic acid (Ni-NTA) agarose resin (Beyotime Biotechnology, Shanghai, China) for 8 h at 4°C under gentle shaking. The recombinant protein was purified by standard Ni-NTA affinity chromatography. The His6 tag was removed by incubation with thrombin for 4 h at 37°C. The purity and concentration of the ANT(6)-If protein was validated by SDS-PAGE and the BCA protein assay kit (Thermo Fisher Scientific, Rockford, IL, United States). The quaternary structure of ANT(6)-If was examined by clear-native PAGE. Two proteins with isoelectric points close to that of the ANT(6)-If (33.7 kDa, pI: 4.4), bovine serum albumin (BSA, 66.4 kDa, pI: 4.7) and ovalbumin (45 kDa, pI: 4.6) were selected as the protein marker for clear-native PAGE (Wittig and Schägger, 2005). ANT(6)-If and the markers were separated by 12% clear-native PAGE without protein denaturant. Electrophoresis was performed at 180 V for 20 min and then at 200 V for 40 min.

### Enzyme kinetic parameter determination

The enzyme kinetic assay was performed as described previously (Kim et al., 2006). The ANT(6)-If activity was measured by coupled enzyme reactions of UDP-glucose pyrophosphorylase, phosphoglucomutase, and glucose-6-phosphate dehydrogenase. The enzyme activity of ANT(6)-If was assayed by monitoring the accumulation of NADPH at 340 nm with a Synergy^™^ Neo2 Multi-Mode Microplate Reader (BioTek Instruments, Inc., United States). The reaction mixture contained 50 mM HEPES (pH 7.5), 10 mM MgCl2, 0.2 mM UDP-glucose, 0.2 mM glucose 1,6-bisphosphate, 0.2 mM NADP, 0.2 mM dithiothreitol, 2 units/mL UDP-glucose pyrophosphorylase, 20 units/mL phosphoglucomutase, 20 units/mL glucose-6-phosphate dehydrogenase, 1 mM ATP, 1.40 × 10^−7^ mM of ANT(6)-If, and variable concentrations of aminoglycoside (5–100 μM) in a total volume of 0.2 mL. Reactions were initiated by the addition of ANT(6)-If.

### Whole genome sequencing and bioinformatic analysis

The genomic DNA was sequenced on the Illumina NovaSeq in the 2 × 150 bp paired-end mode and PacBio RS II platforms (Shanghai Personal Biotechnology Co., Ltd., Shanghai, China). The complete genome was initially assembled by Trycycler (Wick et al., 2021) and Flye (Kolmogorov et al., 2019) using PacBio long reads. Then the Illumina short reads were mapped to the genome using BWA (Li and Durbin, 2009), and the quality of the genome was improved by Polypolish (Wick and Holt, 2022). ORFs were predicted by Prokka (Seemann, 2014). Promoter regions of genes were predicated by BPROM (Solovyev, 2011). The translated protein sequences were searched against the NCBI nr database (Sayers et al., 2021), the Swiss-Prot database (Boutet et al., 2016), and the Comprehensive Antibiotic Resistance Database (CARD; McArthur et al., 2013) using DIAMOND blastp (Buchfink et al., 2021). The 16S rRNA gene and pairwise dDDH analysis were performed on the TYGS platform (Meier-Kolthoff and Göker, 2019). The whole-genome average nucleotide identity and genome-to-genome distance were calculated by FastANI (Jain et al., 2018) and Mash (Ondov et al., 2016) in the gcType species identification pipeline (Shi et al., 2020), respectively. ANI was proposed as the gold standard for the prokaryotic species classification, and a recommended threshold of 95% could be used to circumscribe species boundary (Richter and Rosselló-Móra, 2009). The genome distance is an estimate of the overall similarity between two genomes (Auch et al., 2010). The circular genome map was drawn by CGView Comparison Tool (Grant et al., 2012). The molecular weight and isoelectric point (pI) of protein sequences were calculated by EMBOSS pepstats (Rice et al., 2000). The maximum-likelihood tree was reconstructed, tested and visualized by IQ-TREE (Nguyen et al., 2015), UFBoot2 (Hoang et al., 2018), and ggtree (Yu, 2020), respectively. The multiple sequence alignment was achieved through MAFFT (Katoh and Standley, 2013) and presented by R package msa (Bodenhofer et al., 2015). The synteny figure of genes was drawn by the clinker (Gilchrist and Chooi, 2021). Entrez Direct^3^ and GNU Parallel (Tange, 2021) were used to access the NCBI databases. Samtools (Li et al., 2009) and SeqKit (Shen et al., 2016) were used to manipulate sequence data.

## Results and discussion

### Genome characterization of PATH554

**Figure 1:**
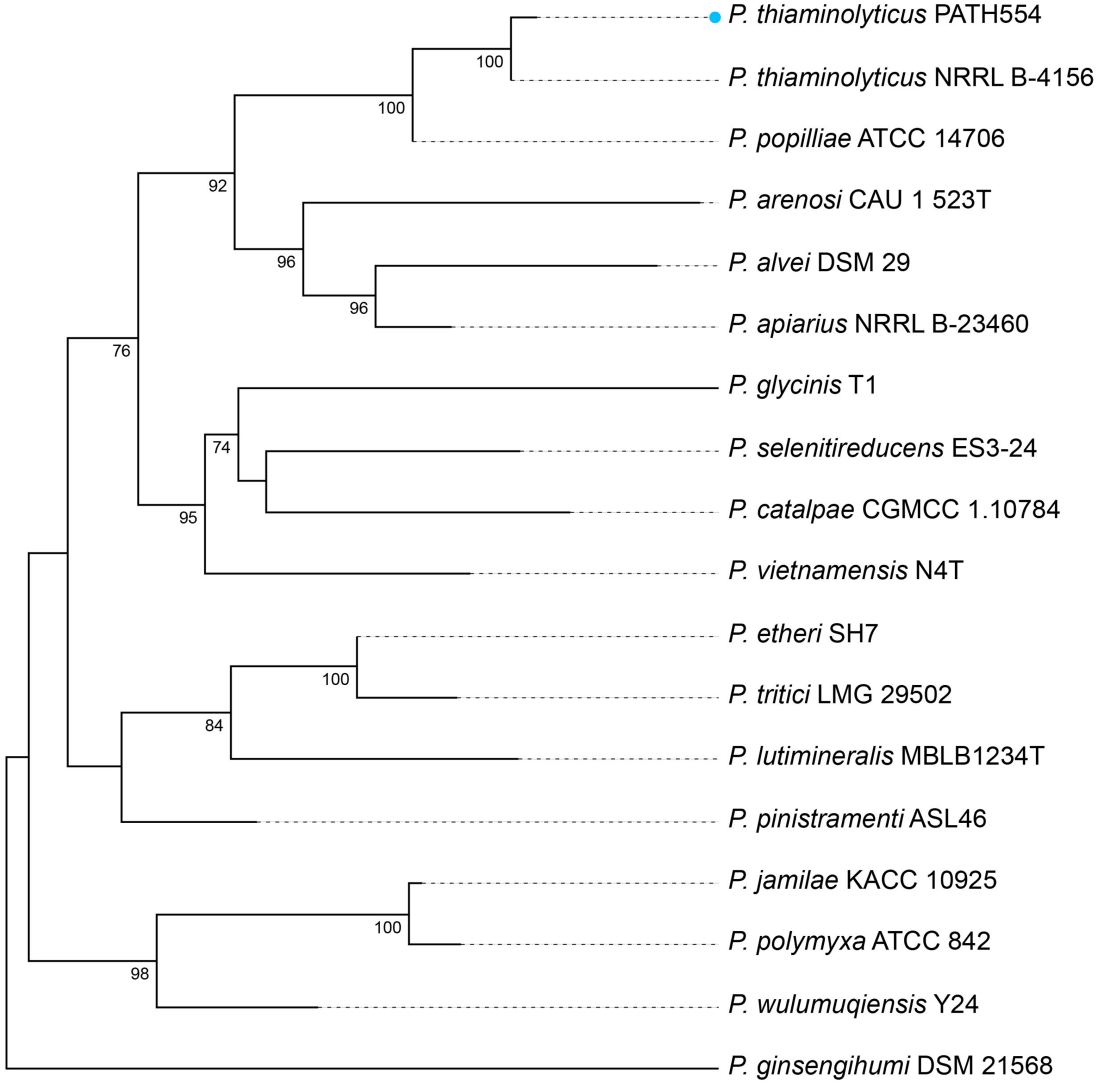
Phylogram based on 16S rRNA genes. *P. thiaminolyticus* PATH554 in this work (blue dot) was phylogenetically close to *P. thiaminolyticus* NRRL B-4156. The numbers near the nodes represented the corresponding bootstrap value.

*P. thiaminolyticus* PATH554 harbored one 6,485,805 bp chromosome (CP114031.1) with a GC content of 53.67%, encoding 5,743 proteins, 83 tRNAs, and 24 rRNAs (Table 4). No plasmid was carried by PATH554. To identify the taxonomic classification of PATH554, we combined the results of 16S rRNA gene, dDDH and ANI analyses. Phylogram based on the 16S rRNA gene indicated that PATH554 is phylogenetically close to *P. thiaminolyticus* (Figure 1). Pairwise dDDH values of PATH554 and other type strains showed that PATH554 was most similar (with 74.4% similarity) to *P. thiaminolyticus* NRRL B-4156 (CP041405.1). PATH554 also shared the highest whole-genome ANI value (97.08%) and minimal genome-to-genome distance (0.0268) against *P. thiaminolyticus* NRRL B-4156. Based on the taxonomic identification results above, PATH554 could be classified into species *P. thiaminolyticus* and thus designated *P. thiaminolyticus* PATH554.

**Table 4:** Genome features of *Paenibacillus thiaminolyticus* PATH554.

Attribute	Value
Title	*P. thiaminolyticus* PATH554
Accession no.	CP114031.1
Molecule type	Genomic DNA
Coverage (×)	1,052
Topology	Circular
Size (bp)	6,485,805
GC content (%)	53.67
CDS	5,743
Known protein	3,288
Hypothetical protein	2,455
Protein coding (%)	98.2
Average protein length	314
tRNA	83
rRNA	24

**Figure 2:**
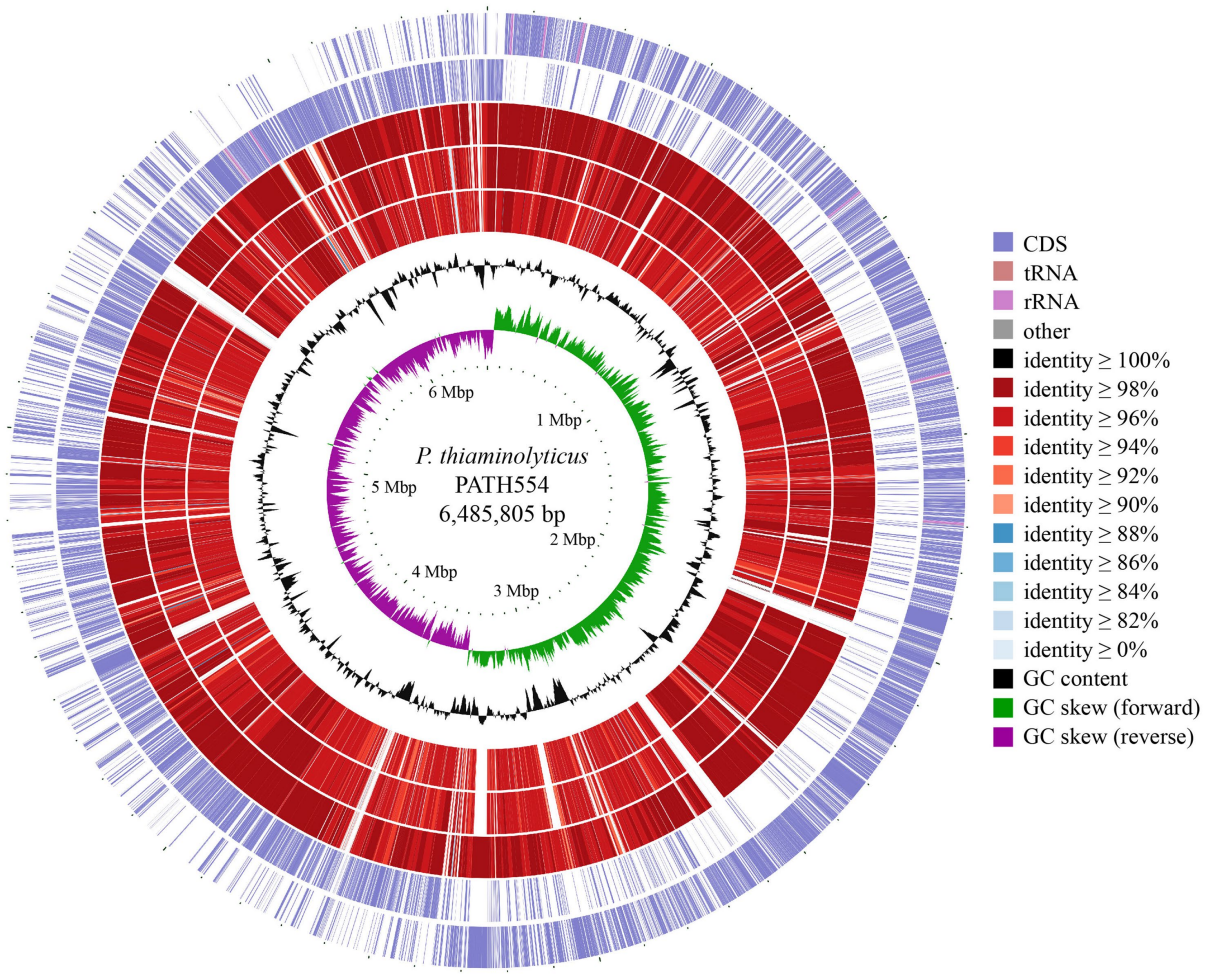
Genome map of the *P. thiaminolyticus* PATH554 and its relatives. Circles 1–7 from outside to inside indicated forward and reverse strand of the *P. thiaminolyticus* PATH554 chromosome (this work), the chromosomes of *P. thiaminolyticus* SY20 (CP106992.1), *P. thiaminolyticus* NRRL B-4156 (CP041405.1), and *P. thiaminolyticus* Mbale2 (CP094446.1), GC content and GC skew, respectively. The saturation of color on circles 3–5 was proportional to blast identity value and the darker color indicated higher sequence similarity and vice versa.

To compare the chromosome of PATH554 and other bacterial genomes, comparative genomic analysis was performed by blastn against the NCBI non-redundant nucleotide database. Up to date, besides PATH554 of this work, 23 other *P. thiaminolyticus* genome assemblies were available in the NCBI assembly database, but only three of them were the complete genomes, which included *P. thiaminolyticus* NRRL B-4156 (CP041405.1), *P. thiaminolyticus* SY20 (CP106992.1) and *P. thiaminolyticus* Mbale2 (CP094446.1). The circular map of PATH554 with the three close relatives was depicted in Figure 2. The complete genome of PATH554 sequenced in this work contributed to the nucleotide database of *P. thiaminolyticus* and would be beneficial for future genome-wide molecular studies of this species.

### Resistance profiles of PATH554 and functional analysis of the novel aminoglycoside resistance gene *ant(6)-if*

By annotating the genome of PATH554 against the CARD database, a total of eight antimicrobial resistance genes (seven genotypes) with ≥ 95% amino acid sequence coverage and ≥ 80% identity were found (Table 5), which showed resistance to three classes of antimicrobials, including one fluoroquinolone resistance gene (*norB*), three lincosamide resistance genes (two copies of *clbB* and one *llmA*), and four glycopeptide resistance genes *vanR*, *vanH*, *vanF* and *vanX*.

**Table 5:** Antimicrobial resistance genes encoded on the chromosome of *Paenibacillus thiaminolyticus* PATH554.

Resistance genes	Function
*norB*	fluoroquinolone resistance gene
*llmA*	lincosamide resistance gene
clbB*2	lincosamide resistance gene
*vanR*	glycopeptide resistance related gene
*vanH*	glycopeptide resistance related gene
*vanF*	glycopeptide resistance related gene
*vanX*	glycopeptide resistance related gene

When analyzing the resistance mechanism, we found that none of the eight genes was associated with the resistance to aminoglycoside antimicrobials, even though this strain showed high MIC levels to several aminoglycosides tested (Table 2), such as tobramycin (16 μg/mL), streptomycin (32 μg/mL), ribostamycin (64 μg/mL) and kanamycin (64 μg/mL). The phenomenon indicated the antimicrobial resistance might be mediated by unknown mechanisms. To figure out whether there was a putative aminoglycoside resistance gene encoded in the PATH554 genome, we checked the annotation result of the genome and found 251 putative antimicrobial resistance genes with <80% identity present, including some predicted aminoglycoside resistance genes, such as *aadK-and aph(3′)-*like. One of the predicted genes showed the highest amino acid homology (100% coverage and 75.35% identity) with the function characterized aminoglycoside modifying enzyme AadK (CAB14620.1) that contributed resistance to streptomycin (N et al., 1993). To determine the potential resistance function of the gene, we cloned the ORF of this *aadK*-like gene with its promoter region into the pMD19 vector, and it was confirmed to be functional. According to the nomenclature proposed for genes encoding aminoglycoside modifying enzymes (Ramirez and Tolmasky, 2010), the novel resistance gene of this work was designated *ant(6)-If* (OP970560.1). The recombinant (pMD19-*ant(6)-If*/DH5α) showed increased MIC levels (by approximately 128-fold) for streptomycin compared with the control strains (DH5α or DH5α carrying pMD19). No significant increase in the MIC value of any other antimicrobial tested was observed (Table 2).

Currently, there were 5 function-characterized ANT(6)s in the CARD database. They were ANT(6)-Ia (AHE40557.1), ANT(6)-Ib (CBH51824.1), AadK (CAB14620.1), AadS (AAA27459.1) and AAD(6) (AAU10334.1). Although they all conferred resistance to streptomycin alone (Kono et al., 1987; Smith et al., 1992; Schwarz et al., 2001; Abril et al., 2010; Yang et al., 2014), they showed different MIC levels against streptomycin. The transconjugant with a transferred plasmid pRE25 carrying an *aad(6)* gene resulted a > 5-fold increase of MIC value for streptomycin compared with that of the recipient (Schwarz et al., 2001). Similar to *ant(6)-If*, *ant(6)-Ia* also showed a high resistance level against streptomycin and the *E. coli* strain with the recombinant plasmid carrying *ant(6)-Ia* increased >216-fold of the MIC level for streptomycin compared to the recipient *E. coli* carrying vector pUC19 only (Yang et al., 2014), while *ant(6)-Ib* on the shuttle vector plasmid pCA75 mediated only a 5-fold increase to streptomycin (Abril et al., 2010).

Furthermore, the enzyme was over-expressed (Supplementary Figure S1) and purified (Supplementary Figure S2). The enzymatic parameters of ANT(6)-If showed a *k*_cat_ and *K*_m_ of 1.12 × 10^−1^ and 1.24 × 10^−5^, respectively, and a *k*_cat_*/K*_m_ ratio of 9.01 × 10^3^ M^−1^·s^−1^ (Supplementary Figure S3). The enzymological properties of ANT(6)-If also showed difference compared to AadK. The *K*_m_ of AadK was 8.0 × 10^−8^ M (Kono et al., 1987), which means a higher binding affinity than that of ANT(6)-If of this work (1.24 × 10^−5^ M).

The inactivation of streptomycin could be catalyzed by ANT(6) through the transfer of the AMP group from ATP to position six of the streptidine moiety (Latorre et al., 2016). In the streptomycin recognition process, positions 2, 3, and 4 in the streptose unit, position 1 in the glucose ring and positions 1 and 6 in the streptidine moiety, are in close contact with the ANT(6) protein binding residue, while positions 3–6 of the glucose unit play a minor role (Corzana et al., 2005). ANT(6)-If may act in the same way.

### Comparative analysis of ANT(6)-If with other ANTs

**Figure 3:**
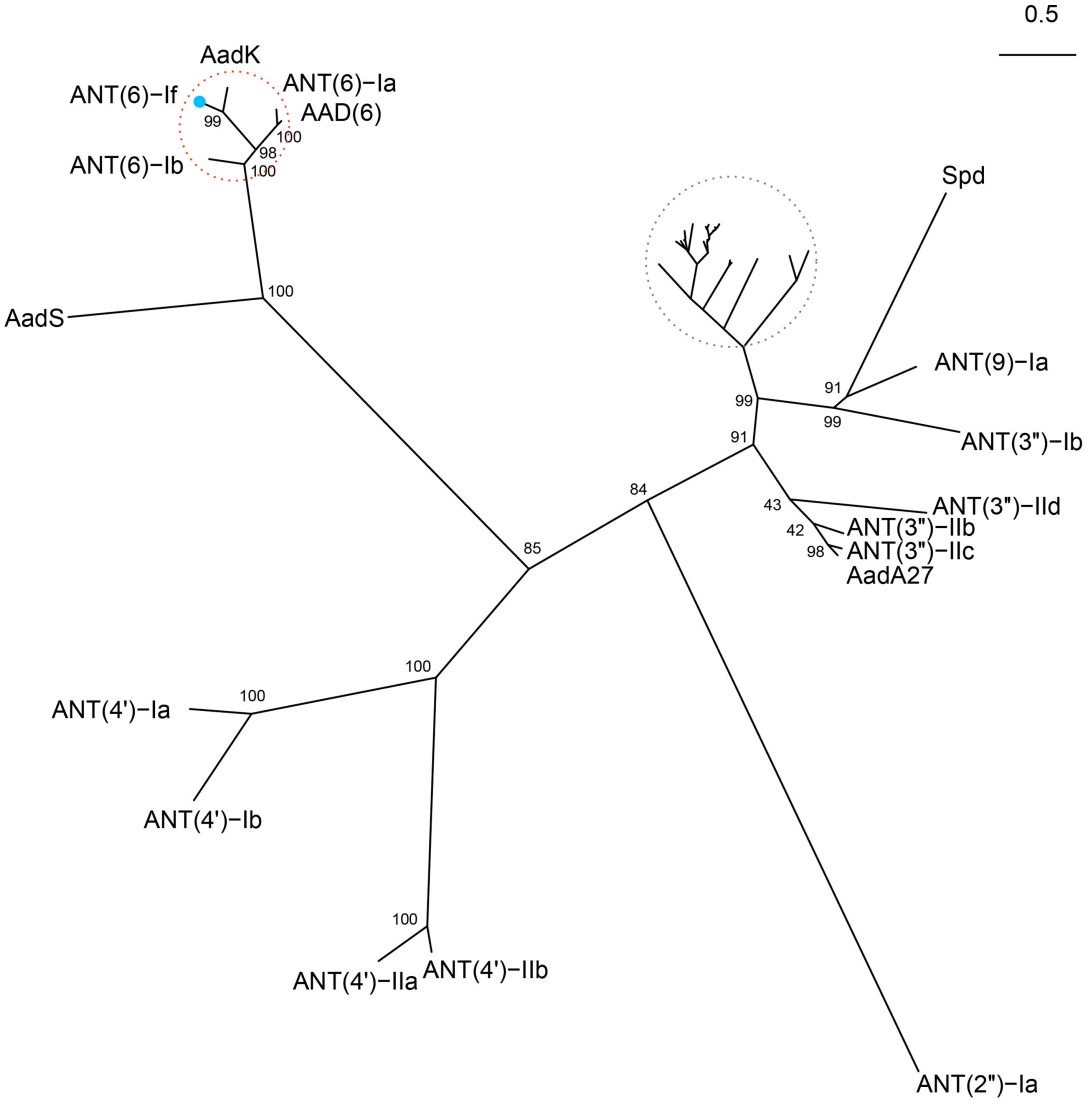
Unrooted phylogenetic tree of ANT(6)-If and other function-characterized ANTs. ANT(6)-If (this work) was on the branch of ANT(6) family cluster located at the top-left corner. The ANT(6) cluster was highlighted with a red circle and ANT(6)-If was indicated by a blue dot. Accession numbers of the ANTs were AadK (CAB14620.1), ANT(6)-Ia (AHE40557.1), AAD(6) (AAU10334.1), ANT(6)-Ib (CBH51824.1), AadS (AAA27459.1), ANT(4′)-Ia (AAO83986.1), ANT(4′)-Ib (ADA62098.1), ANT(4′)-IIa (AAA25717.1), ANT (4′)-IIb (AAM76670.1), ANT(2″)-Ia (AAC64365.1), AadA27 (CTQ57092.1), ANT (3″)-IIc (ENU37733.1), ANT(3″)-IIb (ENU91137.1), ANT(3″)-IId (QUX80205.1), ANT(3″)-Ib (QEQ43477.1), ANT(9)-Ia (CAA26428.1), and Spd (AGW81558.1). Those ANTs in the grey circle were not labeled due to the high density. They included AadA (AAO49597.1), AadA10 (AAL36430.1), AadA11 (AAV32840.1), AadA12 (ACJ47200.1), AadA13 (ABW91178.1), AadA14 (CAI57696.1), AadA15 (ABD58917.1), AadA16 (ACF17980.1), AadA17 (ACK43806.1), AadA2 (AAF27727.1), AadA21 (AAN87151.1), AadA22 (CAK12750.1), AadA23 (CAH10847.1), AadA24 (ABG72894.1), AadA25 (AET15272.1), AadA3 (AAC14728.1), AadA4 (AAN34365.1), AadA5 (AAF17880.1), AadA6 (CAJ32504.1), AadA6/AadA10 (CAJ32491.1), AadA7 (BAD00739.1), AadA8 (AAN41439.1), AadA8b (CAJ13568.1), AadA9 (ABG49324.1), AadA33 (UVE15953.1), AadA36 (UVE15954.1), ANT(3″)-IIa (CAA26199.1), and ANT(3″)-Ii-AAC(6′)-Iid (AAL51021.2).

The 855 bp *ant(6)-If* gene had a less common start codon TTG (Blattner et al., 1997). The protein ANT(6)-If encoded by *ant(6)-If* contained 284 amino acid, with a molecular weight of 33.678 kDa and pI of 4.44. By searching against the CARD database, ANT(6)-If only showed higher similarities with 5 sequences from the ANT(6) family mentioned above. Among them, AadK exhibited the highest amino acid sequence similarity with 100% coverage and 75.35% identity, and then were ANT(6)-Ia (97% coverage and 55.44% identity), ANT(6)-Ib (100% coverage and 54.93% identity), AAD(6) (88% coverage and 57.20% identity), and AadS (94% coverage and 25.91% identity). To figure out the phylogenetic relationship of ANT(6)-If with the other function-characterized ANTs, the phylogenetic tree based on maximum likelihood algorithm was reconstructed. ANT(6)-If located in the branch composed of ANT(6)s, and it indicated that ANT(6)-If was closely related with other ANT(6)s and belonged to the ANT(6) family (Figure 3).

The structure of AadK was determined by X-ray (2PBE). It contains fourteen helixes and eight beta strands, and the structural conservation of ANT(6)-If and the other ANT(6) enzymes (Supplementary Figure S4) was analyzed. Some residues were fully conserved across ANT(6) enzymes, e.g., 44D and 45I in the second beta strand, and 61 W in the third helix. The results of clear-native PAGE showed that the size of ANT(6)-If was between 45 kDa and 66 kDa (Supplementary Figure S5), which suggested that ANT(6)-If was likely to be a homodimer.

**Figure 4:**
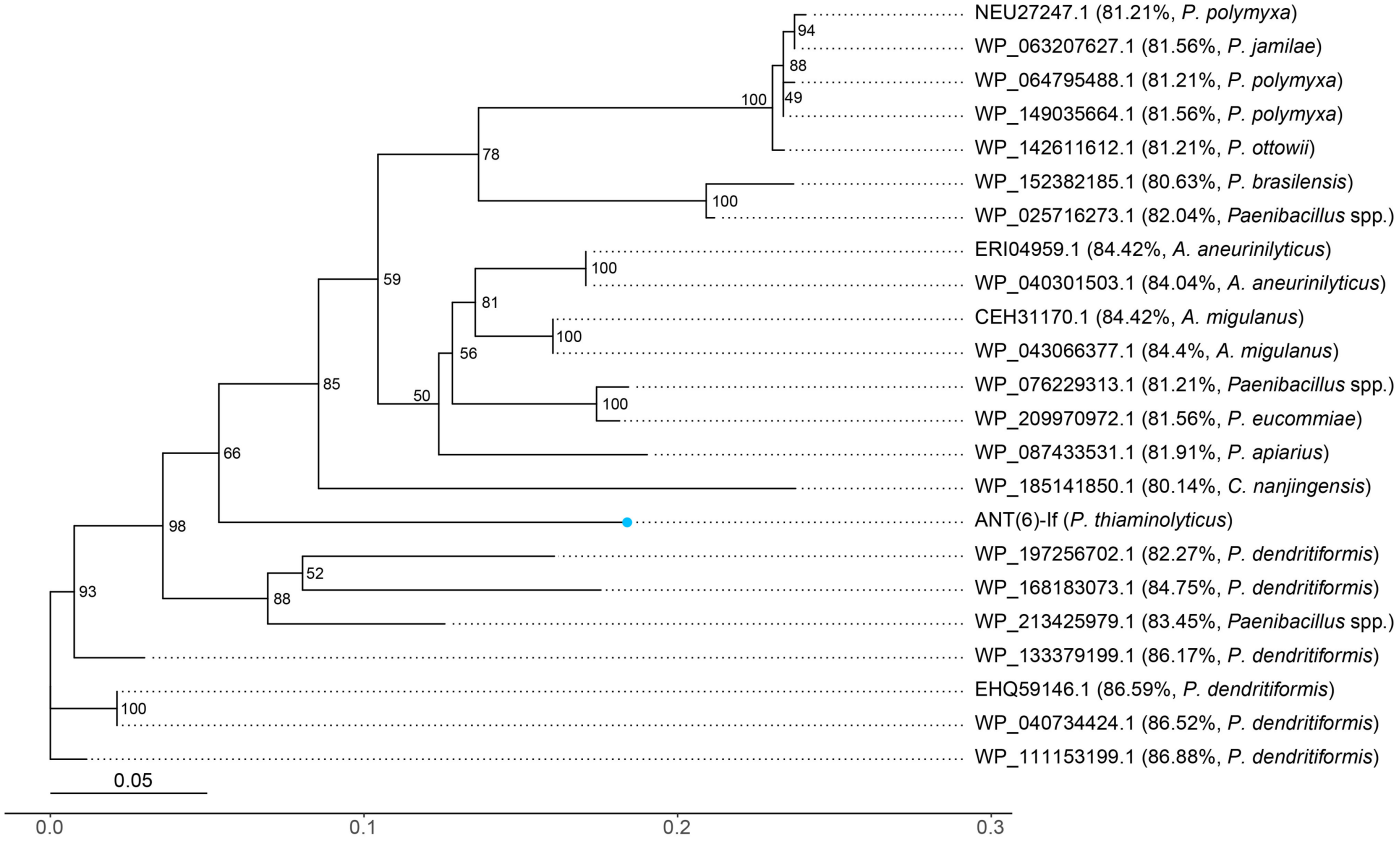
Unrooted phylogenetic tree of ANT(6)-If and other putative ANTs. The numbers near the nodes represented the corresponding bootstrap value. ANT(6)-If (this work) was indicated by a blue dot. The putative ANTs were indicated by their accession numbers. The percentage number and species name represented the amino acid identity of that sequence against ANT(6)-If and the taxonomy of that sequence, respectively.

The distribution of other ANT(6)-If-like ANTs in the NCBI database was also investigated (Figure 4). Among the 22 ANT(6)-If-like ANTs sharing ≥95% coverage and ≥ 80% identity with ANT(6)-If, most (17/22, 77.3%) of them were from genus *Paenibacillus*, and the rest were from genus *Aneurinibacillus* (4/22, 18.2%) or *Cohnella* (1/22, 4.5%). The three genera *Paenibacillus*, *Aneurinibacillus* and *Cohnella* were all endospore-forming bacteria belonging to family *Paenibacillaceae*.

### Synteny analysis of the *ant(6)-If* related sequence

**Figure 5:**
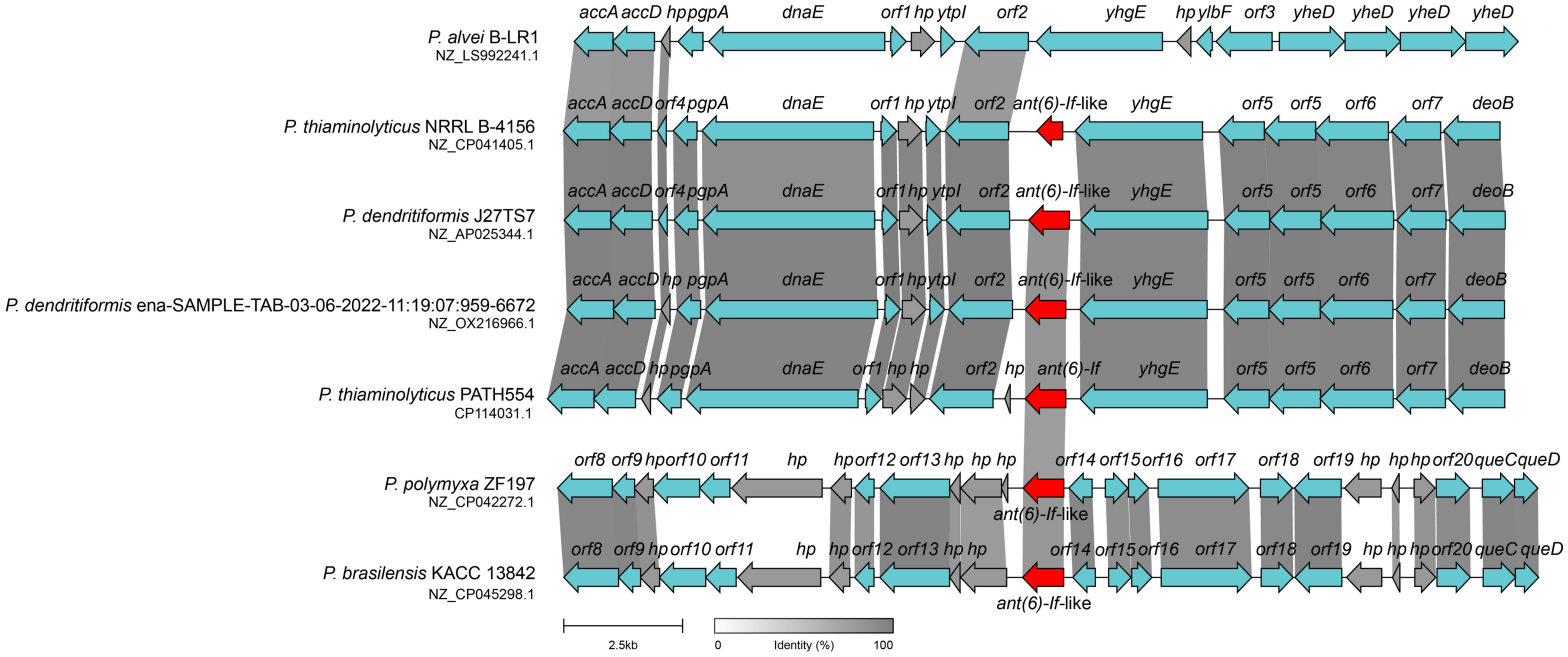
Synteny analysis of *ant(6)-If* and *ant(6)-If*-like genes located on the chromosomes. The *ant(6)-If* (this work) and *ant(6)-If*-like genes were highlighted in red. *hp*: hypothetical protein. Regions shared ≥80% amino acid identities were shaded grey. Accession numbers: *P. alvei* B-LR1 (NZ_LS992241.1), *P. thiaminolyticus* NRRL B-4156 (NZ_CP041405.1), *P. dendritiformis* J27TS7 (NZ_AP025344.1), *P. dendritiformis* ena-SAMPLE-TAB-03-06-2022-11:19:07:959–6,672 (NZ_OX216966.1), *P. thiaminolyticus* PATH554 (CP114031.1), *P. polymyxa* ZF197 (NZ_CP042272.1) and *P. brasilensis* KACC 13842 (NZ_CP045298.1).

Both Gram-negative and Gram-positive bacteria could capture or disseminate antimicrobial resistance genes through the action of mobile genetic elements. Thus, it is a challenging health care problem that bacteria susceptible to antibiotics became resistant through such mechanism (Partridge et al., 2018). When analyzing the synteny of the *ant(6)-If* related sequences, we examined the 20 kbp chromosomal fragment containing the *ant(6)-If* gene and compared it with other sequences in the NCBI non-redundant nucleotide database. The mobile genetic element (MGE) like an insertion sequence (IS), transposon or integron was not found at the flanking region of *ant(6)-If* or *ant(6)-If*-like genes. The structure of the *ant(6)-If* related sequence of *P. thiaminolyticus* PATH554 was similar to those corresponding sequences of *P. thiaminolyticus* NRRL B-4156 (NZ_CP041405.1, 93.3% identity), *P. dendritiformis* J27TS7 (NZ_AP025344.1, 88.5% identity), and *P. dendritiformis* ena-SAMPLE-TAB-03-06-2022-11:19:07:959–6,672 (NZ_OX216966.1, 89.7% identity). Genes encoded at the upstream regions of the *ant(6)-If* and *ant(6)-If*-like genes were those encoding acetyl coenzyme A carboxylase, phosphatidylglycerophosphatase A and DNA polymerase III subunit alpha etc., and genes at the downstream regions were ABC transporter permease, ABC transporter ATP-binding protein and so on. Although the chromosome sequences of *P. polymyxa* ZF197 (NZ_CP042272.1) and *P. brasilensis* KACC 13842 (NZ_CP045298.1) contained *ant(6)-If*-like genes, the flanking regions of the *ant(6)-If*-like genes were drastically different from that of PATH554 (Figure 5). Unlike *ant(6)-If* and *ant(6)-If-like* genes analyzed in this work, other *ant(6)* genes has been found to be related with MGEs. *aadK* was found on both the chromosome (Kono et al., 1987) and the conjugative multi-resistance plasmid pRE25 which was reported to be able to integrate into the chromosome (Schwarz et al., 2001). *aadS* was found to locate on a transposon Tn*4551* (Smith et al., 1992). *ant(6)-Ia* was identified on a plasmid pMC1 (Yang et al., 2014) and Tn*5405* (Denapaite et al., 2010), and *ant(6)-Ib* was within a transferable pathogenicity island on a chromosome (Abril et al., 2010) and Tn*1806* (Corver et al., 2012).

## Conclusion

In this study, we identified a novel aminoglycoside resistance gene *ant(6)-If* which located in a relatively conserved genomic region on the chromosome of *Paenibacillus thiaminolyticus* PATH554 and it encoded an aminoglycoside *O*-nucleotidyltransferase ANT(6)-If. The enzyme ANT(6)-If belonged to the ANT(6) family and shared the highest amino acid identity with the function-characterized aminoglycoside *O*-nucleotidyltransferase AadK. ANT(6)-If conferred resistance to streptomycin with the *k*_cat_*/K*_m_ of 9.01 × 10^3^ M^−1^·s^−1^. Identification of the novel resistance gene helped to better understand the complex resistance mechanisms in bacterial population.

## Data availability statement

The datasets generated for this study can be found in the NCBI GenBank under accession numbers OP970560.1 (*ant(6)-If*) and CP114031.1 (the chromosome of *P. thiaminolyticus* PATH554).

## Ethics statement

This study used strains obtained from an anal swab of a rabbit on an animal farm in Wenzhou. The owner of the farm was informed of the study and expressed approval for sampling of animals. All experimental procedures involving animals were approved by the Animal Welfare and Ethics Committee of Wenzhou Medical University, Zhejiang Province, China (Animal protocol number: wydw2021-0323).

## Author contributions

XL, QB, and CF: conceived and designed the experiments. MG, JL, WS, XL, and KL: performed the experiments. XL, MG, JL, and CF: data analysis and interpretation. QB and CF: drafting of the manuscript. All authors contributed to the article and approved the submitted version.

## Funding

This study was supported by the Science and Technology Project of Jinhua City, China (2022-2-013 and 2022-4-017), the Science and Technology Project of Wenzhou City, China (N20210001) and Zhejiang Provincial Natural Science Foundation of China (LY19C060002 and LQ17H190001).

## Conflict of interest

The authors declare that the research was conducted in the absence of any commercial or financial relationships that could be construed as a potential conflict of interest.

## Publisher’s note

All claims expressed in this article are solely those of the authors and do not necessarily represent those of their affiliated organizations, or those of the publisher, the editors and the reviewers. Any product that may be evaluated in this article, or claim that may be made by its manufacturer, is not guaranteed or endorsed by the publisher.

## Footnotes

^1^https://lpsn.dsmz.de/genus/paenibacillus

^2^https://www.snapgene.com/

^3^https://ftp.ncbi.nlm.nih.gov/entrez/entrezdirect/

## Supplementary material

Supplementary Figure S1 Analysis of the recombinant protein expression by SDS-PAGE. Lane 1: sediment; lane 2: supernatant; lane M: PageRuler Prestained Protein Ladder (10 to 180 kDa).

Supplementary Figure S2 Analysis of the recombinant protein purity by SDS-PAGE. Lane FT: flow through; lane W1, W4 and W6: wash 1, 4 and 6; lane M: PageRuler Prestained Protein Ladder (10 to 180 kDa); lane E1-E10: elution 1-10.

Supplementary Figure S3 Reaction rate as a function of streptomycin concentration. The concentration of streptomycin was 5, 10, 15, 25, and 50 μM/L, respectively.

Supplementary Figure S4 Multiple sequence alignment of ANT(6)-If (this work) and the other ANT(6)s. The secondary structures were marked on the top. The length of each sequence was displayed on the right. Asterisks and exclamations indicated highly and fully conserved residues, respectively. The logo size at the bottom indicated the degree of conservation of residues. Accession numbers: AadK (CAB14620.1), ANT(6)-Ia (AHE40557.1), ANT(6)-Ib (CBH51824.1), AAD(6) (AAU10334.1), and AadS (AAA27459.1).

Supplementary Figure S5 Clear-native PAGE of ANT(6)-If. Lane 1: ANT(6)-If (this work); lane 2: bovine serum albumin (BSA); lane 3: PageRuler Prestained Protein Ladder (10 to 180 kDa); lane 4: ovalbumin.

## References

[ref1] Abril C. Brodard I. Perreten V. (2010). Two novel antibiotic resistance genes, tet(44) and ant(6)-Ib, are located within a transferable Pathogenicity Island in *Campylobacter fetus* subsp. fetus. Antimicrob. Agents Chemother. 54, 3052–3055. doi: 10.1128/AAC.00304-10, PMID: 20479200 PMC2897286

[ref2] Auch A. F. Klenk H.-P. Göker M. (2010). Standard operating procedure for calculating genome-to-genome distances based on high-scoring segment pairs. Stand. Genomic Sci. 2, 142–148. doi: 10.4056/sigs.541628, PMID: 21304686 PMC3035261

[ref3] Becker B. Cooper M. A. (2013). Aminoglycoside antibiotics in the 21st century. ACS Chem. Biol. 8, 105–115. doi: 10.1021/cb3005116 23110460

[ref4] Blattner F. R. Plunkett G. Bloch C. A. Perna N. T. Burland V. Riley M.. (1997). The complete genome sequence of *Escherichia coli* K-12. Science 277, 1453–1462. doi: 10.1126/science.277.5331.1453, PMID: 9278503

[ref5] Bodenhofer U. Bonatesta E. Horejš-Kainrath C. Hochreiter S. (2015). Msa: an R package for multiple sequence alignment. Bioinformatics 31, 3997–3999. doi: 10.1093/bioinformatics/btv494, PMID: 26315911

[ref6] Boutet E. Lieberherr D. Tognolli M. Schneider M. Bansal P. Bridge A. J.. (2016). UniProtKB/Swiss-Prot, the manually annotated section of the UniProt KnowledgeBase: how to use the entry view. Methods Mol. Biol. 1374, 23–54. doi: 10.1007/978-1-4939-3167-5_2, PMID: 26519399

[ref7] Buchfink B. Reuter K. Drost H.-G. (2021). Sensitive protein alignments at tree-of-life scale using DIAMOND. Nat. Methods 18, 366–368. doi: 10.1038/s41592-021-01101-x, PMID: 33828273 PMC8026399

[ref8] Corver J. Bakker D. Brouwer M. S. M. Harmanus C. Hensgens M. P. Roberts A. P.. (2012). Analysis of a *Clostridium difficile* PCR ribotype 078 100 kilobase island reveals the presence of a novel transposon, Tn6164. BMC Microbiol. 12:130. doi: 10.1186/1471-2180-12-130, PMID: 22747711 PMC3485107

[ref9] Corzana F. Cuesta I. Bastida A. Hidalgo A. Latorre M. González C.. (2005). Molecular recognition of aminoglycoside antibiotics by bacterial defence proteins: NMR study of the structural and conformational features of streptomycin inactivation by *Bacillus subtilis* aminoglycoside-6-adenyl transferase. Chemistry 11, 5102–5113. doi: 10.1002/chem.200400941, PMID: 15984036

[ref10] Denapaite D. Brückner R. Nuhn M. Reichmann P. Henrich B. Maurer P.. (2010). The genome of *Streptococcus mitis* B6- what is a commensal? PLoS One 5:e9426. doi: 10.1371/journal.pone.0009426, PMID: 20195536 PMC2828477

[ref11] Di Micco R. Schneider M. Nüesch R. (2021). Postoperative *Paenibacillus thiaminolyticus* wound infection, Switzerland. Emerg. Infect. Dis. 27, 1984–1986. doi: 10.3201/eid2707.203348, PMID: 34152975 PMC8237901

[ref12] Gilchrist C. L. M. Chooi Y.-H. (2021). Clinker & clustermap.Js: automatic generation of gene cluster comparison figures. Bioinformatics 37, 2473–2475. doi: 10.1093/bioinformatics/btab007 33459763

[ref13] Grady E. N. MacDonald J. Liu L. Richman A. Yuan Z.-C. (2016). Current knowledge and perspectives of *Paenibacillus*: a review. Microb. Cell Factories 15:203. doi: 10.1186/s12934-016-0603-7, PMID: 27905924 PMC5134293

[ref14] Grant J. R. Arantes A. S. Stothard P. (2012). Comparing thousands of circular genomes using the CGView comparison tool. BMC Genomics 13:202. doi: 10.1186/1471-2164-13-202, PMID: 22621371 PMC3469350

[ref15] Hoang D. T. Chernomor O. von Haeseler A. Minh B. Q. Vinh L. S. (2018). UFBoot2: improving the ultrafast bootstrap approximation. Mol. Biol. Evol. 35, 518–522. doi: 10.1093/molbev/msx281, PMID: 29077904 PMC5850222

[ref16] Hormeño L. Ugarte-Ruiz M. Palomo G. Borge C. Florez-Cuadrado D. Vadillo S.. (2018). Ant(6)-I genes encoding aminoglycoside O-Nucleotidyltransferases are widely spread among streptomycin resistant strains of campylobacter jejuni and *Campylobacter coli*. Front. Microbiol. 9:2515. doi: 10.3389/fmicb.2018.02515, PMID: 30405573 PMC6206021

[ref17] Huang E. Yousef A. E. (2014). Paenibacterin, a novel broad-spectrum lipopeptide antibiotic, neutralises endotoxins and promotes survival in a murine model of *Pseudomonas aeruginosa*-induced sepsis. Int. J. Antimicrob. Agents 44, 74–77. doi: 10.1016/j.ijantimicag.2014.02.018, PMID: 24802906

[ref18] Jain C. Rodriguez-R L. M. Phillippy A. M. Konstantinidis K. T. Aluru S. (2018). High throughput ANI analysis of 90K prokaryotic genomes reveals clear species boundaries. Nat. Commun. 9:5114. doi: 10.1038/s41467-018-07641-9, PMID: 30504855 PMC6269478

[ref19] Katoh K. Standley D. M. (2013). MAFFT multiple sequence alignment software version 7: improvements in performance and usability. Mol. Biol. Evol. 30, 772–780. doi: 10.1093/molbev/mst010, PMID: 23329690 PMC3603318

[ref9001] Kim C. Hesek D. Zajícek J. Vakulenko S. B. Mobashery S. (2006). Characterization of the bifunctional aminoglycoside-modifying enzyme ANT(3’’)-Ii/AAC(6’)-IId from Serratia marcescens. Biochemistry 45, 8368–8377. doi: 10.1021/bi060723g, PMID: 16819836

[ref20] Kolmogorov M. Yuan J. Lin Y. Pevzner P. A. (2019). Assembly of long, error-prone reads using repeat graphs. Nat. Biotechnol. 37, 540–546. doi: 10.1038/s41587-019-0072-8, PMID: 30936562

[ref21] Kono M. Ohmiya K. Kanda T. Noguchi N. O’hara K. (1987). Purification and characterization of chromosomal streptomycin adenylyltransferase from derivatives of *Bacillus subtilis* Marburg 168. FEMS Microbiol. Lett. 40, 223–228. doi: 10.1111/j.1574-6968.1987.tb02029.x

[ref22] Latorre M. Revuelta J. García-Junceda E. Bastida A. (2016). 6-O-Nucleotidyltransferase: an aminoglycoside-modifying enzyme specific for streptomycin/streptidine. Med. Chem. Commun. 7, 177–183. doi: 10.1039/C5MD00496A

[ref23] Li H. Durbin R. (2009). Fast and accurate short read alignment with burrows-wheeler transform. Bioinformatics 25, 1754–1760. doi: 10.1093/bioinformatics/btp324, PMID: 19451168 PMC2705234

[ref24] Li H. Handsaker B. Wysoker A. Fennell T. Ruan J. Homer N.. (2009). The sequence alignment/map format and SAMtools. Bioinformatics 25, 2078–2079. doi: 10.1093/bioinformatics/btp352, PMID: 19505943 PMC2723002

[ref25] McArthur A. G. Waglechner N. Nizam F. Yan A. Azad M. A. Baylay A. J.. (2013). The comprehensive antibiotic resistance database. Antimicrob. Agents Chemother. 57, 3348–3357. doi: 10.1128/AAC.00419-13, PMID: 23650175 PMC3697360

[ref26] Meier-Kolthoff J. P. Auch A. F. Klenk H.-P. Göker M. (2013). Genome sequence-based species delimitation with confidence intervals and improved distance functions. BMC Bioinformatics 14:60. doi: 10.1186/1471-2105-14-60, PMID: 23432962 PMC3665452

[ref27] Meier-Kolthoff J. P. Göker M. (2019). TYGS is an automated high-throughput platform for state-of-the-art genome-based taxonomy. Nat. Commun. 10:2182. doi: 10.1038/s41467-019-10210-3, PMID: 31097708 PMC6522516

[ref28] Morton S. U. Hehnly C. Burgoine K. Ssentongo P. Ericson J. E. Kumar M. S.. (2022). *Paenibacillus* infection causes neonatal sepsis and subsequent postinfectious hydrocephalus in ugandan infants.

[ref29] N N. M S. M K. (1993). Genetic mapping in *Bacillus subtilis* 168 of the aadK gene which encodes aminoglycoside 6-adenylyltransferase. FEMS Microbiol. Lett. 114, 47–52. doi: 10.1016/0378-1097(93)90140-w, PMID: 8293959

[ref30] Nguyen L.-T. Schmidt H. A. von Haeseler A. Minh B. Q. (2015). IQ-TREE: a fast and effective stochastic algorithm for estimating maximum-likelihood phylogenies. Mol. Biol. Evol. 32, 268–274. doi: 10.1093/molbev/msu300, PMID: 25371430 PMC4271533

[ref31] Ondov B. D. Treangen T. J. Melsted P. Mallonee A. B. Bergman N. H. Koren S.. (2016). Mash: fast genome and metagenome distance estimation using min hash. Genome Biol. 17:132. doi: 10.1186/s13059-016-0997-x, PMID: 27323842 PMC4915045

[ref32] Ouyang J. Pei Z. Lutwick L. Dalal S. Yang L. Cassai N.. (2008). Case report: *Paenibacillus thiaminolyticus*: a new cause of human infection, inducing bacteremia in a patient on hemodialysis. Ann. Clin. Lab. Sci. 38, 393–400. PMID: 18988935 PMC2955490

[ref33] Partridge S. R. Kwong S. M. Firth N. Jensen S. O. (2018). Mobile genetic elements associated with antimicrobial resistance. Clin. Microbiol. Rev. 31, e00088–e00017. doi: 10.1128/CMR.00088-17, PMID: 30068738 PMC6148190

[ref34] Qing G. Ma L.-C. Khorchid A. Swapna G. V. T. Mal T. K. Takayama M. M.. (2004). Cold-shock induced high-yield protein production in *Escherichia coli*. Nat. Biotechnol. 22, 877–882. doi: 10.1038/nbt984, PMID: 15195104

[ref35] Ramirez M. S. Tolmasky M. E. (2010). Aminoglycoside modifying enzymes. Drug Resist. Updat. 13, 151–171. doi: 10.1016/j.drup.2010.08.003, PMID: 20833577 PMC2992599

[ref36] Rice P. Longden I. Bleasby A. (2000). EMBOSS: the European molecular biology open software suite. Trends Genet. 16, 276–277. doi: 10.1016/s0168-9525(00)02024-2, PMID: 10827456

[ref37] Richter M. Rosselló-Móra R. (2009). Shifting the genomic gold standard for the prokaryotic species definition. Proc. Natl. Acad. Sci. U. S. A. 106, 19126–19131. doi: 10.1073/pnas.0906412106, PMID: 19855009 PMC2776425

[ref38] Richter C. A. Wright-Osment M. K. Zajicek J. L. Honeyfield D. C. Tillitt D. E. (2009). Quantitative polymerase chain reaction (PCR) assays for a bacterial thiaminase I gene and the thiaminase-producing bacterium *Paenibacillus thiaminolyticus*. J. Aquat. Anim. Health 21, 229–238. doi: 10.1577/H07-054.1, PMID: 20218497

[ref39] Sáez-Nieto J. A. Medina-Pascual M. J. Carrasco G. Garrido N. Fernandez-Torres M. A. Villalón P.. (2017). *Paenibacillus* spp. isolated from human and environmental samples in Spain: detection of 11 new species. New Microbes New Infect 19, 19–27. doi: 10.1016/j.nmni.2017.05.006, PMID: 28702198 PMC5484988

[ref40] Sayers E. W. Bolton E. E. Brister J. R. Canese K. Chan J. Comeau D. C.. (2021). Database resources of the National Center for biotechnology information. Nucleic Acids Res. 50, D20–D26. doi: 10.1093/nar/gkab1112, PMID: 34850941 PMC8728269

[ref41] Schwarz F. V. Perreten V. Teuber M. (2001). Sequence of the 50-kb conjugative multiresistance plasmid pRE25 from *Enterococcus faecalis* RE25. Plasmid 46, 170–187. doi: 10.1006/plas.2001.1544, PMID: 11735367

[ref42] Seemann T. (2014). Prokka: rapid prokaryotic genome annotation. Bioinformatics 30, 2068–2069. doi: 10.1093/bioinformatics/btu153, PMID: 24642063

[ref43] Shen W. Le S. Li Y. Hu F. (2016). SeqKit: a cross-platform and ultrafast toolkit for FASTA/Q file manipulation. PLoS One 11:e0163962. doi: 10.1371/journal.pone.0163962, PMID: 27706213 PMC5051824

[ref44] Shi W. Sun Q. Fan G. Hideaki S. Moriya O. Itoh T.. (2020). gcType: a high-quality type strain genome database for microbial phylogenetic and functional research. Nucleic Acids Res. 49, D694–D705. doi: 10.1093/nar/gkaa957, PMID: 33119759 PMC7778895

[ref45] Shida O. Takagi H. Kadowaki K. Nakamura L. K. Komagata K. (1997). Transfer of *Bacillus alginolyticus*, *Bacillus chondroitinus*, *Bacillus curdlanolyticus*, *Bacillus glucanolyticus*, Bacillus kobensis, and *Bacillus thiaminolyticus* to the genus *Paenibacillus* and emended description of the genus *Paenibacillus*. Int. J. Syst. Bacteriol. 47, 289–298. doi: 10.1099/00207713-47-2-289, PMID: 9103612

[ref46] Smith C. J. Owen C. Kirby L. (1992). Activation of a cryptic streptomycin-resistance gene in the Bacteroides erm transposon, Tn4551. Mol. Microbiol. 6, 2287–2297. doi: 10.1111/j.1365-2958.1992.tb01404.x, PMID: 1328814

[ref47] Solovyev V. (2011). “Automatic annotation of microbial genomes and metagenomic sequences,” in Metagenomics and its applications in agriculture, biomedicine and environmental studies. ed. Li R. W. (Nova Science Publishers), 61–78.

[ref48] Tange O. (2021). GNU Parallel 20210822 (“Kabul”). Zenodo:120. doi: 10.5281/zenodo.5233953

[ref49] Wachino J.-I. Doi Y. Arakawa Y. (2020). Aminoglycoside resistance: updates with a focus on acquired 16S ribosomal RNA Methyltransferases. Infect. Dis. Clin. N. Am. 34, 887–902. doi: 10.1016/j.idc.2020.06.002 PMC10927307 33011054

[ref50] Wang N. Luo J. Deng F. Huang Y. Zhou H. (2022). Antibiotic combination therapy: a strategy to overcome bacterial resistance to aminoglycoside antibiotics. Front. Pharmacol. 13:839808. doi: 10.3389/fphar.2022.839808, PMID: 35281905 PMC8905495

[ref51] Wick R. R. Holt K. E. (2022). Polypolish: short-read polishing of long-read bacterial genome assemblies. PLoS Comput. Biol. 18:e1009802. doi: 10.1371/journal.pcbi.1009802, PMID: 35073327 PMC8812927

[ref52] Wick R. R. Judd L. M. Cerdeira L. T. Hawkey J. Méric G. Vezina B.. (2021). Trycycler: consensus long-read assemblies for bacterial genomes. Genome Biol. 22:266. doi: 10.1186/s13059-021-02483-z, PMID: 34521459 PMC8442456

[ref53] Wittig I. Schägger H. (2005). Advantages and limitations of clear-native PAGE. Proteomics 5, 4338–4346. doi: 10.1002/pmic.200500081, PMID: 16220535

[ref54] Wu Y. Liu D. Liang M. Huang Y. Lin J. Xiao L. (2022). Genome-guided purification and characterization of polymyxin A1 from *Paenibacillus thiaminolyticus* SY20: a rarely explored member of polymyxins. Front. Microbiol. 13:962507. doi: 10.3389/fmicb.2022.962507, PMID: 36452932 PMC9701815

[ref55] Yang J. Wang C. Wu J. Liu L. Zhang G. Feng J. (2014). Characterization of a multiresistant mosaic plasmid from a fish farm sediment Exiguobacterium sp. isolate reveals aggregation of functional clinic-associated antibiotic resistance genes. Appl. Environ. Microbiol. 80, 1482–1488. doi: 10.1128/AEM.03257-13, PMID: 24362420 PMC3911065

[ref56] Yu G. (2020). Using ggtree to visualize data on tree-like structures. Curr. Protoc. Bioinformatics 69:e96. doi: 10.1002/cpbi.96, PMID: 32162851

