# Supplementary figures and images for: Identification and characterization of a novel aminoglycoside O-nucleotidyltransferase ANT(6)-If from Paenibacillus thiaminolyticus PATH554
Source: Front Microbiol. 2023 Jun 29;14:1184349. doi: 10.3389/fmicb.2023.1184349 (PMC10343464; doi:10.3389/fmicb.2023.1184349)

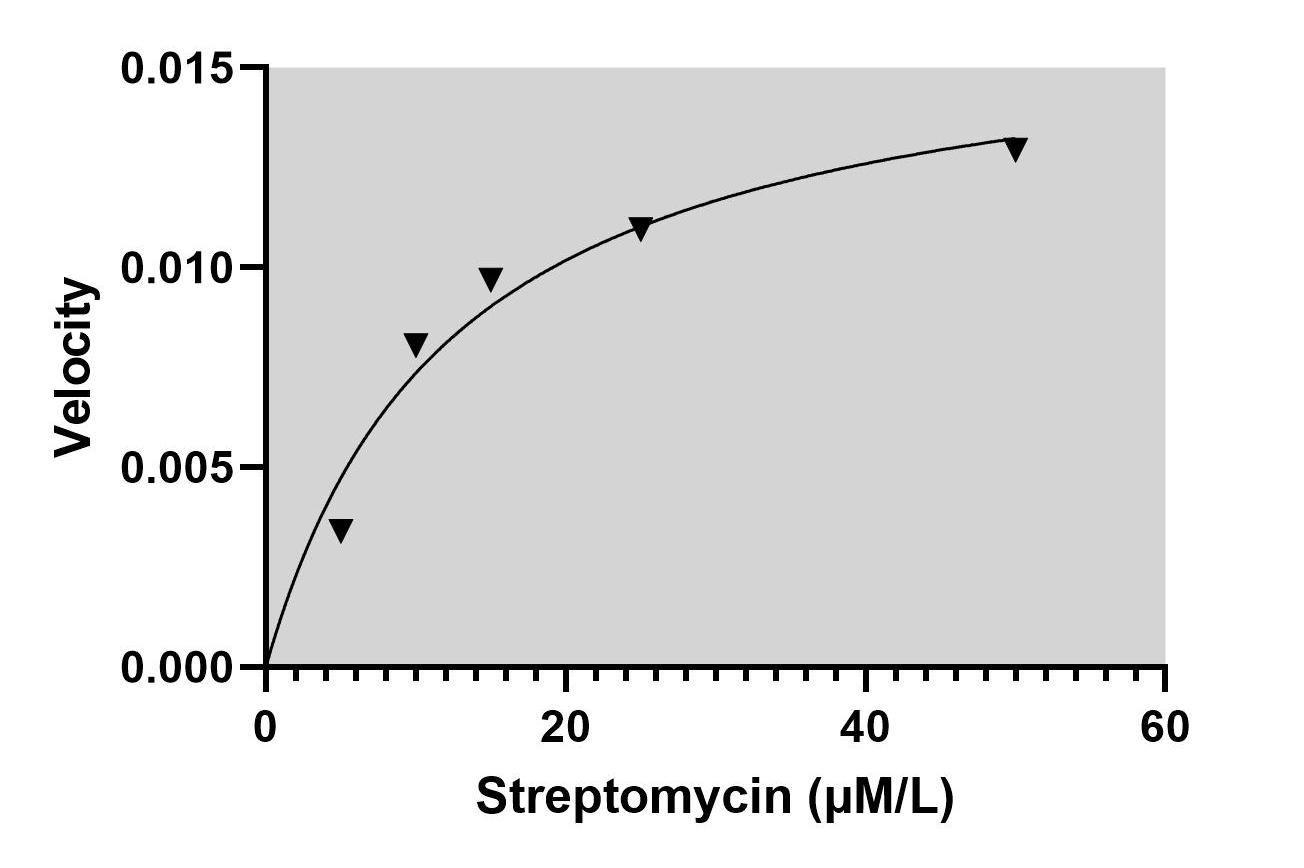

Supplement: Supplementary Figure S3 — Reaction rate as a function of streptomycin concentration. The concentration of streptomycin was 5, 10, 15, 25, and 50 μM/L, respectively. [file Image_3.JPEG]

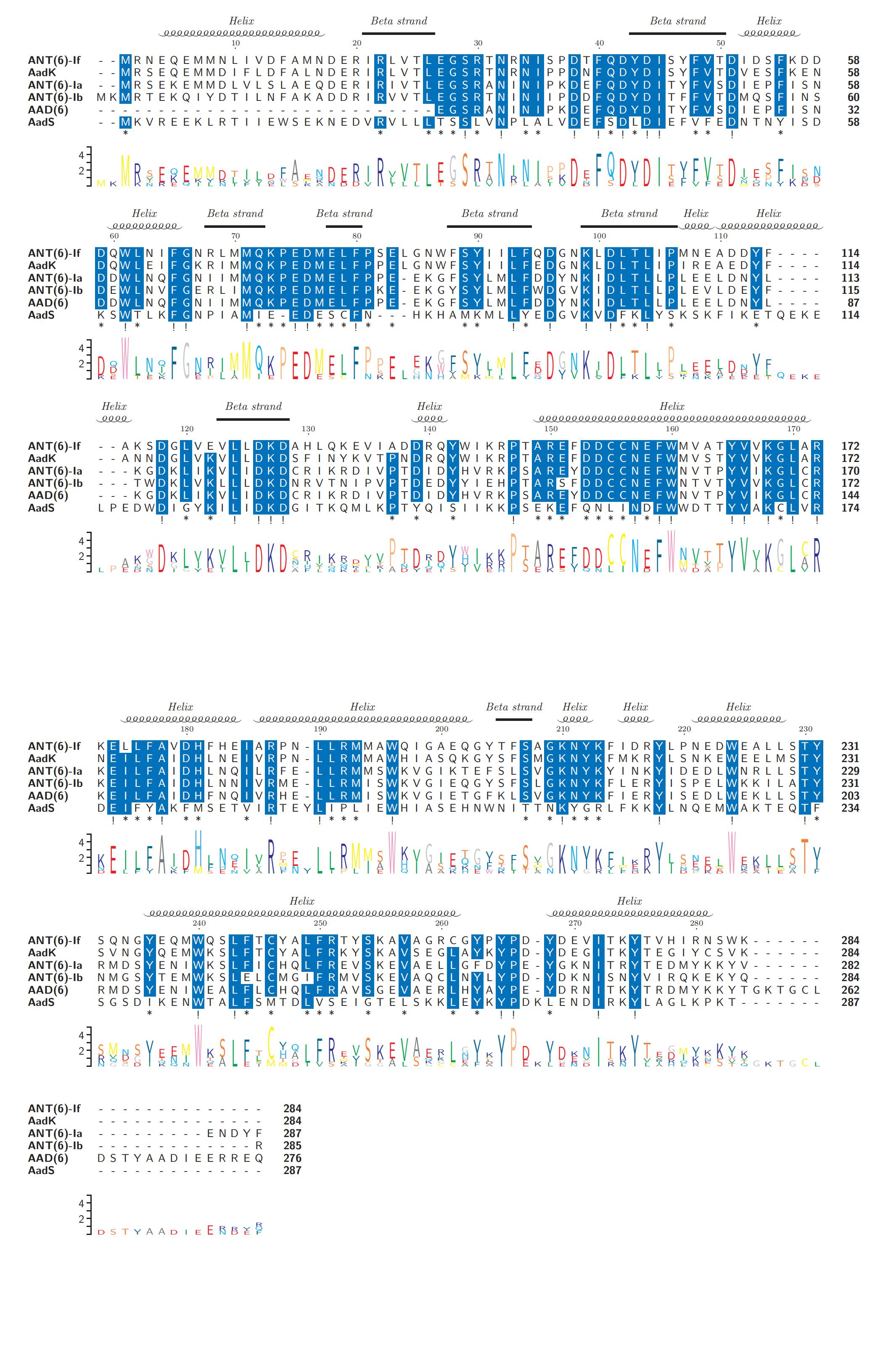

Supplement: Supplementary Figure S4 — Multiple sequence alignment of ANT(6)-If (this work) and the other ANT(6)s. The secondary structures were marked on the top. The length of each sequence was displayed on the right. Asterisks and exclamations indicated highly and fully conserved residues, respectively. The logo size at the bottom indicated the degree of conservation of residues. Accession numbers: AadK (CAB14620.1), ANT(6)-Ia (AHE40557.1), ANT(6)-Ib (CBH51824.1), AAD(6) (AAU10334.1), and AadS (AAA27459.1). [file Image_4.JPEG]

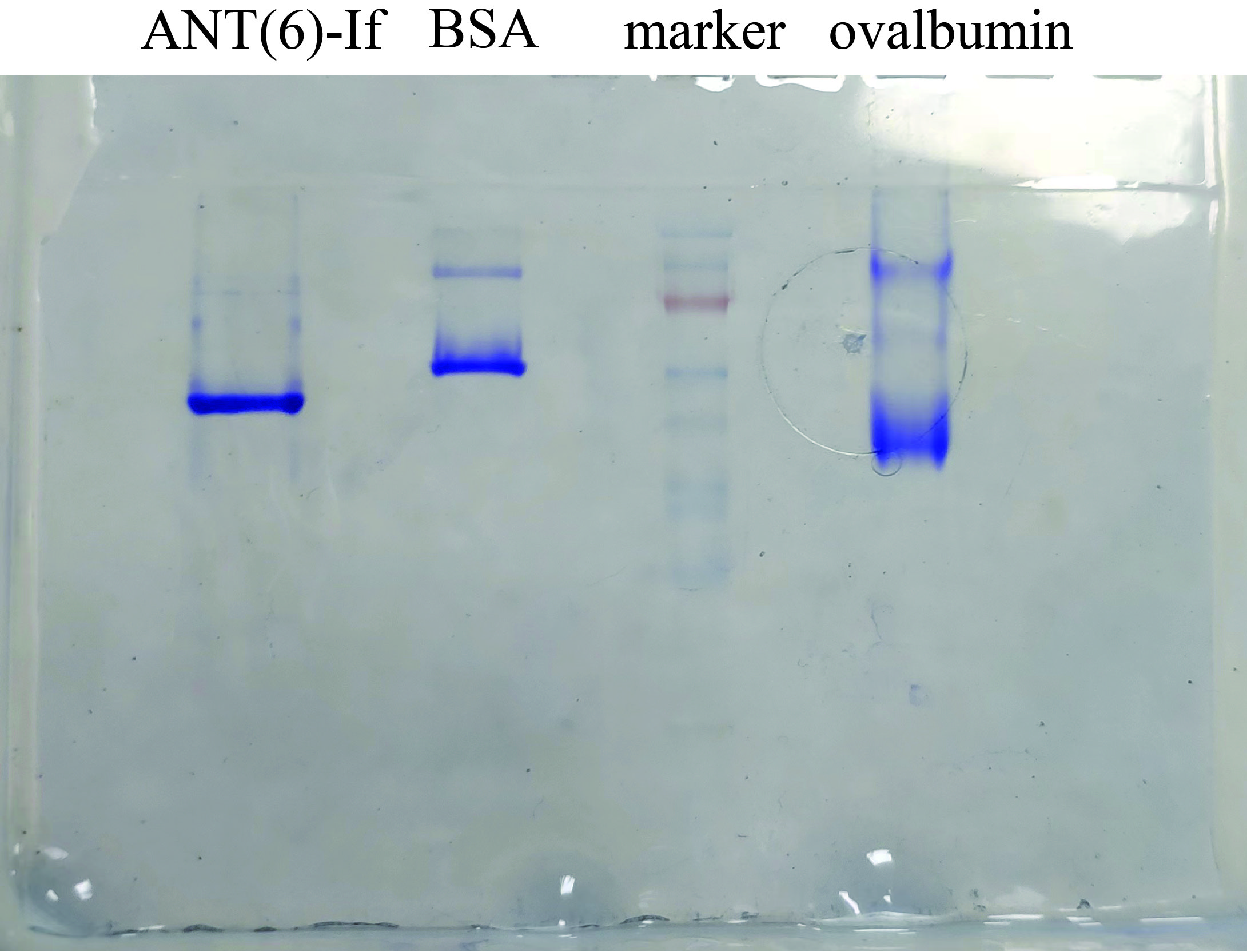

Supplement: Supplementary Figure S5 — Clear-native PAGE of ANT(6)-If. Lane 1: ANT(6)-If (this work); lane 2: bovine serum albumin (BSA); lane 3: PageRuler Prestained Protein Ladder (10 to 180 kDa); lane 4: ovalbumin. [file Image_5.JPEG]
